# The Ongoing “Doctor–Nurse Game”: Power, Knowledge, and Advocacy Through the Lens of Nursing Standpoint Theory

**DOI:** 10.1111/nin.70129

**Published:** 2026-07-08

**Authors:** Kathleen Shearer, Steven Hall

**Affiliations:** ^1^ Faculty of Nursing, Edmonton Clinic Health Academy University of Alberta Edmonton Alberta Canada

**Keywords:** advocacy, critical theory, healthcare systems, nurse–patient relationship, nursing standpoint theory, patient‐centered care, power dynamics

## Abstract

There is ongoing tension in healthcare where certain types of knowledge are privileged above others to the detriment of patient outcomes. This hierarchy marginalizes nursing knowledge and patient perspectives, reinforcing systemic inequities in care delivery. Revisiting Stein's (1967) “doctor–nurse game,” in which nurses were expected to influence care indirectly while preserving physician authority, this paper examines how hierarchical patterns of communication and knowledge recognition continue to shape practice. Nursing standpoint theory serves as a framework for critically examining power dynamics within the healthcare setting, providing a means to critically examine how power operates within healthcare structures and interprofessional relationships. Applying nursing standpoint theory reveals how systemic power imbalances shape provider interactions and affect the ability of nurses to advocate for patients. The analysis demonstrates that nurses' embeddedness in patient care positions them uniquely to recognize and respond to gaps in care. Understanding the ways nurses and physicians are situated in relation to power helps explain persistent tensions and offers a path toward more equitable care delivery. Rather than treating advocacy as an individual reactive task, centering the nursing standpoint reframes it as a relational and structural practice that depends on reciprocal recognition of professional knowledge. Empowering nurses to navigate and reshape power relations is therefore essential to improving outcomes. This theoretical reorientation benefits patients, teams, and health systems by shifting the “game” from deference to deliberation in support of patient‐centered care.

## 1 Background

Despite decades of professional change, the nurse–physician relationship remains shaped by hierarchical power dynamics that influence how knowledge is recognized and how patient advocacy is carried out in practice. As we seek to improve the services provided, increase efficiencies within and across health systems, and responsibly attend to rising healthcare expenditures, constrained resources, and ongoing allocation pressures, it is imperative that nurses do not lose sight of patients and their unique needs. Nursing is a *values‐based profession*, and as such, nurses must be committed to providing high‐quality, humane, and respectful patient care (S. M. Scott and Scott 2021). Nursing expertise and contributions to knowledge development significantly shape patient health outcomes, yet systemic constraints, including chronic understaffing and resource allocation challenges, continue to disempower nurses and limit their influence on healthcare systems.

There is a longstanding tension in healthcare where certain types of knowledge are privileged above others with consequences for patient outcomes. In his seminal article “The Doctor–Nurse Game,” Leonard I. Stein (1967) examined the hidden dynamics of communication and authority between doctors and nurses within hospital settings. Stein (1967) argued that, while nurses often made valuable clinical recommendations, they were expected to do so in an indirect and deferential manner, suggesting ideas without appearing to challenge the doctor's authority. This delicate interplay, which he likened to a “game,” was governed by strict, unspoken rules designed to preserve the physician's sense of control while still allowing nurses to contribute meaningfully to patient care. Stein (1967) critiqued this interaction as inefficient and psychologically unhealthy, rooted in gender roles, hierarchical training, and professional insecurities. He concluded that while the system often functioned smoothly, it inhibited open dialogue and mutual respect and ultimately called for a cultural shift toward more direct and collaborative communication between healthcare professionals. Contemporary manifestations may be subtle rather than overt, including differential uptake of recommendations, expectations of indirect communication, or unequal recognition of clinical judgment.

This discursive piece discusses power dynamics, situated within the patient advocacy paradigm, examined through the nursing lens, to critically examine a historically entrenched, formally challenged, yet still persistent model of physician‐led hierarchy. In this paper, nursing standpoint theory serves as the theoretical framework for a critical examination of power dynamics in healthcare settings. Drawing on Risjord's (2011) nursing standpoint theory, we show how nurses' positionality, shaped by these dynamics, situates them as privileged knowers who enhance patient advocacy. In doing so, the thread of power, which winds its way throughout the healthcare system, is examined in our discussion, ultimately highlighting effective collaboration strategies to address our patients' diverse health and social needs. It is important to note that we do not look to suggest that all nurse–physician relationships are oppositional, nor that hierarchical dynamics are experienced uniformly across settings. Rather, in this work, we argue that standpoint theory remains useful for examining how such dynamics can still shape advocacy and knowledge recognition in practice, reframing the role that nurses, and nursing as a way of knowing, can play in the healthcare sphere.

## 2 Nursing Standpoint Theory

Nursing standpoint theory derives from standpoint theory and draws from the feminist perspective (Longino 1993). Standpoint epistemology maintains that four conditions must be met for a viewpoint to be considered privileged (Risjord 2011):

1. it is oppressed by the dominant role,
2. it is structured by the needs and interests of the dominant role,
3. it has practices that are invisible to those in the dominant role, and
4. the role is understood specifically from their own viewpoint in addition to that of their oppressor.

Risjord (2011) illustrated how nursing standpoint differs from traditional standpoint theorists in that nursing values are at the core of the profession and practice of nursing, whereas social justice is central to the latter. Reed expanded standpoint theory by arguing that it is “epistemically advantageous when the practitioner of science is also a practitioner of nursing” (2022, 43). This underscores that nurses' experiential and scientific knowledge together provide a privileged epistemic standpoint. By scientific knowledge, we do not mean only biomedical or laboratory‐based forms of knowing. In this paper, we use the term broadly to include empirically informed clinical reasoning while also recognizing that nursing knowledge may be simultaneously evidence‐based, experiential, relational, and contextual. This perspective directly relates to power differentials, as it positions nursing as distinct and authoritative within healthcare. Our argument is not that nursing knowledge should displace medical expertise in all domains, but that different forms of professional knowledge become salient in different moments of care, and that patient advocacy is strengthened when these are engaged reciprocally rather than hierarchically.

## 3 Power Dynamics in Healthcare

The nursing standpoint is a privileged position from which to advocate for patients (Risjord 2011). However, critically appraising it requires understanding the tension created by power dynamics in healthcare relationships. Power dynamics exist between doctors and nurses (Balanon Bocato 2018; D'Antonio 2022; Stein 1967), nurses and patients (Lupton 1995), and between nurses, doctors, and healthcare administrators (Broom et al. 2023). The longstanding power imbalance between nurses and doctors often limits the role nurses can play as advocates for their patients (Butler and Fox 2024). Yet, these relational imbalances are not new. Lupton (1995) explored the power dynamics that exist between doctors and patients, as well as between nurses and patients. She proposed four different definitions of power by Parsons, Weber, Marxists, and Foucault and ultimately settled on the Foucauldian perspective as being the most appropriate. Through this lens, Foucault described power as a strategy which supports “objectification” of the patient and argued that power differentials and “apparent ‘dehumanizing’ may benefit both health care professional and patient” (Lupton 1995, 162). Lupton (1995) posited that nurses exercise power over patients for reasons different from doctors, and that the mere act of having patients speak, and tell their story, incites a power imbalance greater than if patients simply remained silent. Although this was written in 1995, the tendency to undermine the role of the patient in healthcare interactions conflicts with contemporary emphasis on patient engagement, where patients' active engagement in their own health needs is generally encouraged (Hickmann et al. 2022). However, it remains an important contribution for understanding the roots of these enduring power structures.

This in turn sheds light on the doctor–nurse relationship, the characteristics that sustain power imbalances, and offers examples of how nurses can advocate for patients. As Renzetti said in her recently released novel, “the structural scaffolding of power” (2026, 6) must be understood, as we endeavor to strengthen healthcare. Renzetti further quotes Canadian Senator Paulette Senior as eloquently noting that “the structure was created to benefit those who put it together and continue to maintain it” (2026, 13). From our “standpoint” as nurses, we must understand the structure, who built it, and how it is maintained, with the aim of disrupting the status quo as we advocate with and for our patients. We also acknowledge that nurse–physician power relations are not experienced uniformly; they are mediated by intersectional factors such as gender, race, class, seniority, professional role, and organizational or cultural context.

## 4 Historical Roots of Power in Healthcare

Although Florence Nightingale was instrumental in helping define the domain of nursing, she significantly influenced the future directions of the relationship between nurses and doctors by stating that nurses were to help doctors and not be an impediment to their ability to do their work (Risjord 2011). It may be argued that Nightingale was advocating harmony within the relationship between nurses and doctors, allowing for a synergistic liaison between the two groups. She nevertheless did so in a manner that was “self‐consciously constructed in parallel with Victorian gender roles” (Risjord 2011, 69). The path was paved for doctors to assume the dominant role. Although there are pockets of change whereby nurses now assume more autonomy and independence, Bucknall and Thomas's statement that “medicine holds the legally sanctioned monopoly over central tasks like diagnosis and therapeutic measures” (1997, 229) continues to ring true.

The relationship between nurses and doctors has its roots in paternalism. Mid‐nineteenth century healthcare provision such as diagnosis and treatment, fell under the “exclusive prerogative of medical men” (Witz 1992, 72). Women, for their part, were tasked with providing care for the sick and attending to births (Witz 1992). The laws of the time, such as the 1858 Medical (Registration) Act, did not strictly prohibit women from entering the medical profession. However, societal rules of the day, such as women's exclusion from universities and medical schools created a gendered divide that “sounded the death knell for women's participation in healing practices” (Witz 1992, 72). The fallout from these practices meant that women were at the mercy of exclusionary strategies that restricted their access to the skills, knowledge, competencies, and credentials required to move freely within the medical world (and labor markets) and served to further engender healthcare practices of the day. Women found their way to healthcare as nurses, and patriarchal power influences were ubiquitous.

Nurses were considered handmaidens to doctors, with treatment decisions left largely in physicians' hands (Stein 1967). Stein (1967) articulated that the position of doctors at the time was predominantly male, unabashedly omnipotent, and entirely responsible for management of patient treatment. Nurses, almost always female, were expected to take initiative and make recommendations, while simultaneously appearing passive. Although exceptions to this norm existed, particularly in the case of midwives (Thomas 2009), nursing's historical role remained subordinate to doctors, a dynamic nurses became adept at navigating. As D'Antonio (2022) argued, the truth of modern nursing's emergence lay in the idea that women of the time sought out medical knowledge while doctors were seeking knowledgeable women. The interplay between the two required an intricate balancing of roles, with the goal of the game being a delicate dance: doctors sought advice, but never directly, and nurses provided counsel, but only ever indirectly. This involved using the subtle art of suggestion to allow the doctor to continue to occupy his role as omniscient. Although nursing has progressed far beyond these early subservient roles, some echoes of this “doctor–nurse game” remain visible in contemporary healthcare practices.

### 4.1 Power and Foucault

Power dynamics in healthcare, who holds it, how it is yielded, and how it can be used persist, influencing who holds authority and how it is exercised. Meaningful insight along with analyses of the unacknowledged assumptions and metaphors in healthcare practice is enriched by adherents of Foucault's philosophy (Henderson 1994). Foucault contributed significantly to our understanding of power dynamics in healthcare, notably coining the term “medical gaze” (Hancock 2018). The medical gaze is a lens through which “knowledge is produced, is able to penetrate the body, ascertain its true meanings, master its secrets, diagnose, and prescribe treatment” (Hancock 2018, 444). Udod (2008) summarized Foucault's ideas on power, saying that power is not something that one possesses, but rather a relational strategy, simultaneously enabling and constraining. Udod noted that power “requires the cooperation of the subject (the nurse), and this cooperation is, in part, what renders power relations so effective” (2008, 86). This dynamic was evident in Stein's (1967) account of the doctor–nurse game.

Foucault's insights into modern interpretations of nursing and power dynamics shape perceptions of how nurses should advocate for their patients. Jenkins et al. (2022) noted the importance of nurses acting as truth tellers, asserting that this duty is critical to both improvement and saving of patients' lives. They stated that this role can be seen as daring and even revolutionary, a thought echoed in the Gosport Independent Panel, which affirmed that nurses raising initial concerns was “a brave act given the culture at the hospital” (2018, 320). They also noted that there was evidence in the documents that the nurses felt ostracized because of their advocacy (Gosport Independent Panel 2018). The choice to stand back and let the status quo run its course (as evidenced by the Gosport Independent Panel's findings) can have disastrous results. Structural power inequities played a role in preventing patients from getting the care they needed. Developing advocacy skills is critical for nurses who must navigate these layered power structures.

Foucault's contributions to the discourse of power in healthcare support a deeper appreciation of the ways in which information is transformed. For instance, in intensive care settings, nurses collect information about the body through the medical gaze while healthcare organizations ensure that this gaze is maintained, as institutions determine which aspects of the gaze (knowledge) ought to shape healthcare practice (Henderson 1994). Nurses are uniquely situated to understand the power that, for example, lies within the practice of caring for ICU patients. The challenge then is to recognize this power and learn to wield it to advocate on behalf of their patients, for as Rolin highlighted, “even though relations of power do not always involve domination, they function as vehicles of domination when they constrain an individual's or a group's choices in a way that is harmful for the individual or the group” (2009, 219). Nurses must not undervalue their position, as this jeopardizes their ability to meaningfully and effectively advocate for their patients.

## 5 Patient Advocacy

Patient advocacy is a critical component of nurses' profession and a practical method through which nurses can deliver care (Abbasinia et al. 2019). Due to the unique organizational, coordinating, and integrative position that nurses have within the delivery of healthcare services (P. A. Scott 2017), they are ideally situated to advocate on behalf of their patients. Imbued within the concept of patient advocacy are the notions of power and empowerment. When individuals experience vulnerability, powerlessness, or challenging circumstances, they often require advocates to protect their rights, welfare, and basic needs (Negarandeh et al. 2006). Therefore, it is imperative to equip nurses with the necessary skills and competencies to advocate effectively. An integral aspect of any discussion of power structures in healthcare is an understanding of the unique roles of nurses, doctors, and healthcare administrators. Power differentials among these groups are universal, with variations based on legislative and organizational systems that either perpetuate or minimize these differentials. Nurses are at the heart of any discussion of power and have historically bowed to the power of doctors and administrators (Aspinall et al. 2023; Balanon Bocato 2018; Gosport Independent Panel 2018; Stein 1967), while skillfully navigating the system to secure optimal patient care (Bu and Jezewski 2007; Lupton 1995). As prior research has emphasized, advocacy becomes particularly salient in contexts of vulnerability (Bu and Jezewski 2007; Negarandeh et al. 2006).

In their concept analysis of patient advocacy, Abbasinia et al. (2019) found that patient advocacy embodies more than just the provision of support, compassionate care, and empathy. This dynamic concept also includes “safeguarding, valuing, mediating, and championing social justice in the provision of healthcare” (Abbasinia et al. 2019, 146). For their part, Bu and Jezewski (2007) conceptualized patient advocacy by considering its three core attributes: safeguarding patients' autonomy, acting on behalf of patients, and championing social justice in the provision of healthcare. These core attributes are influenced by macrosocial and microsocial antecedents and present the opportunity for nurses to engage in patient advocacy roles. Nurses must be included in patient advocacy efforts both on the macrosocial level (society at large potentially benefits) and at the microsocial level (individual patient well‐being can be positively influenced) due to their unique standpoint, which allows them to do this in ways other professions cannot. As our healthcare systems become more complex, both in terms of working with advanced technologies and working within resource‐constrained settings, the theory proposed in this article helps provide guidance for nurses looking to engage in patient advocacy.

Bu and Jezewski (2007) helped to situate the contention that nurse–doctor relationships need to continue to evolve, specifically when it comes to theories around patient advocacy. There lies great opportunity to build on and expand the relationship between nurses and doctors, and the opportunity to come together to support patients and their unique needs (individual, family, or community‐based) is made possible through the lens of patient advocacy. Nairn (2019) identified the importance of doctors and nurses coming together with greater intentionality to help advocate in situations which patients will flourish or not. He noted that the combination of nurses and doctors working together has the potential to be very powerful as they advocate for change within the administrative sphere (Nairn 2019). Nairn also noted the importance of being aware of the political sphere within which nurses (and doctors) work as “the marketization of care, inequality and a growing authoritarian nationalism” (2019, 7) threaten nursing knowledge generation. Patient advocacy is successful—and flourishes—when doctors and nurses acknowledge that their unique knowledge bases are a strength and that a commitment to *working with* and not just alongside each other will positively affect patient care. This coordination of efforts will strengthen their collective ability to advocate together on behalf of patients, despite constraints that continue to exist within the healthcare system.

Through her work around death and dying, Gadow (1979) drew attention to the distinction between advocacy and paternalism, two concepts present within the context of her research. Power dynamics are evident in authoritative relationships between doctors, nurses, and dying patients as Gadow carefully explains, noting that paternalism is “the use of coercion to provide a good that is not desired by the one it is intended to benefit” (1979, 390). She noted this is directly in opposition to the concept of advocacy which whereby one “positively contributes to … a person's right to self‐determination” (1979, 390) and which allows for the entire complex nature of one's values to be accounted for when faced with making difficult decisions (Gadow). Effective advocacy requires time as nurses learn about the wants, needs, values, and beliefs that have shaped their patients' lives up to the point of their care. Gadow asserted that advocacy occurs only once a thorough self‐examination of one's views and values is complete, for it is “only in this way, when the self is engaged and expressed in its entirety, can a person's decision actually be self‐determined instead of being merely a decision that is not determined by others or, still worse, a decision that is determined by others” (1979, 391). While this account of advocacy is aspirational and may be difficult to enact under contemporary constraints of time, staffing, and institutional pressure, it remains useful as a normative benchmark against which current practice can be critically assessed.

### 5.1 Examples in Practice Across the Life Course

In pediatric care, parents act as principal advocates and become essential members of their child's healthcare team. Sánchez‐Rubio et al. (2021) identified key barriers and strategies that effect parental decision‐making in processes in pediatric intensive care units. Issues such as communication barriers, subordination of parent roles and loss of trust in nurses all serve to negatively affect parents' abilities to positively engage in the healthcare delivery and advocacy activities (Sánchez‐Rubio et al. 2021). Conversely, strategies nurses can employ such as open sharing of information, inclusion of parents in their child's care, professional competence, and assurances of confidentiality all serve to positively influence power dynamics thereby enhancing relationships and advocacy efforts (Sánchez‐Rubio et al. 2021). Medical complexity in pediatric intensive care units causes extreme stress for patients and parents alike. Nurses can help decrease this stress by teaching parents how to integrate normal parenting tasks into their child's care, promoting family‐centered care (Fisk et al. 2022). In addition to decreasing stress, parents and children's anxiety rates decrease, and rates of parents' satisfaction with healthcare increase when parents are given the opportunity to be involved in their hospitalized child's healthcare needs (Çamur and Sarıkaya Karabudak 2021). As critical members of the healthcare team, nurses play a pivotal role in supporting parents in their role as caregivers, and when this is done well, patients and their parents stand to benefit.

In the provision of care with older adults, power dynamics shape the role of nurses in the context of patient advocacy. Unique to geriatric care are notions of stereotyping and ageism (Eliassen 2016). Implicit biases and stereotypes about older adults can diminish their agency, which affects how their needs and preferences are addressed (Eliassen 2016). Nurses can, should, and do advocate for older adults by liaising information between family and friend caregivers, the older adults in care, and the wider healthcare team (Luca et al. 2021). In this role, we protect older adults' rights and support their autonomy (Abbasinia et al. 2019; Luca et al. 2021). In acute geriatric units, nurses comprise most care providers, and their perception of teamwork is closely linked to their job satisfaction and the perceived quality of care delivered (Piers et al. 2019). Moreover, when nurses and physicians collaborate effectively in geriatric care settings, there is a lower turnover retention among nursing staff (Piers et al. 2019). Although power imbalances limit nurses' abilities to advocate for older adults, our role remains vital for ensuring patient‐centered, ethical, and effective care. While nurses are key members of geriatric care teams, their roles and influence can be limited by unclear responsibilities and traditional power structures. Effective interprofessional teamwork, with clearly defined and respected roles, leads to better care for older adults (Piers et al. 2019). However, more research is needed to clarify and optimize these dynamics for the benefit of both older adults and interprofessional care teams.

## 6 Revisiting the “Doctor–Nurse Game”

Revisiting the “doctor–nurse game” every once in a while can be valuable, as was last done in 1990 (Stein et al. 1990). To consider the game through the lens of nursing standpoint theory highlights that what once operated as tacit etiquette is better understood as a power that organizes voice, visibility, and risk across the interprofessional care team. Stein (1967) named a non‐zero‐sum “game” whose cardinal rule was to avoid open disagreement while converting nurses' clinical judgments into physicians' orders; thereby securing cooperation but suppressing frank dialogue and punishing those who broke the script. Seen through the lens of the nursing standpoint, those interactional “rules” are precisely what standpoint alerts us to—they privilege one way of knowing, render the other as supportive rather than generative, and reproduce hierarchical self‐protection over patient‐centered transparency. The Foucauldian medical gaze shows how such rules travel and mutate so that silence can still pass for teamwork.

Patient advocacy, then, is not a detour from Stein but the corrective he implicitly called for: redesigning the game so that nursing knowledge is named, credited, and invited upstream. In this reframed “game,” the win condition is not deference but deliberation: a just culture where nurses act with courage to advocate for patients and physicians are responsive partners who work toward the same goal. The nursing standpoint situated as it is, within the space of oppressed (and potentially oppressor) provides essential comprehension of the innate power structures found within the healthcare setting. Nursing standpoint theory offers a critical framework for analyzing the historical and ongoing power dynamics inherent in the nurse–doctor relationship. A comprehensive understanding of the power relationships among nurses, doctors, and the broader interprofessional team and healthcare system, coupled with targeted strategies to address power imbalances, can empower nurses to more effectively advocate for their patients.

## Funding

The authors have nothing to report.

## Disclosure

No generative artificial intelligence (AI) tools or large language models were used in the drafting, editing, or creation of this manuscript. All research, data analysis, and written content are the original work of the authors, who take full responsibility for their integrity.

## Ethics Statement

The authors have nothing to report.

## Conflicts of Interest

The authors declare no conflicts of interest.

## Data Availability

Data sharing not applicable to this article as no datasets were generated or analyzed during the current study.
